# Characteristics and Long-Term Outcome of 535 Patients with Autoimmune Hepatitis—The 20-Year Experience of a High-Volume Tertiary Center

**DOI:** 10.3390/jcm12134192

**Published:** 2023-06-21

**Authors:** Matthias Buechter, Dominik Dorn, Birte Möhlendick, Winfried Siffert, Hideo A. Baba, Guido Gerken, Alisan Kahraman

**Affiliations:** 1 Department of Gastroenterology and Hepatology, University Clinic of Essen, University of Duisburg-Essen, 45122 Essen, Germany; 2 Department of Gastroenterology and Hepatology, Elisabeth Hospital, 58638 Iserlohn, Germany; 3 Institute of Pharmacogenetics, University Clinic of Essen, University of Duisburg-Essen, 45122 Essen, Germany; 4 Institute of Pathology, University Clinic of Essen, University of Duisburg-Essen, 45122 Essen, Germany; 5 Department of Gastroenterology and Hepatology, Helios Clinic, 42549 Velbert, Germany; 6 Department of Gastroenterology and Hepatology, Max Grundig Clinic, 77815 Bühl, Germany

**Keywords:** autoimmune hepatitis, immunosuppression, liver transplantation

## Abstract

**Background and aims:** Autoimmune hepatitis (AIH) is a complex and progressive inflammatory liver disease characterized by immune-mediated destruction of the liver parenchyma, hypergammaglobulinemia, the presence of circulating autoantibodies, and good response to immunosuppressive therapy. Since the prevalence of AIH is relatively rare, data on the clinical course and the long-term outcome are scarce. **Patients and methods:** We retrospectively analyzed the data of 535 well-documented AIH patients treated at the University Hospital Essen between 2000 and 2020. **Results:** The majority of patients were middle-aged females (75% women, mean age 45 years) with AIH type 1 (97%). Approximately 32% of patients were diagnosed with cirrhosis due to AIH, 29% had concomitant autoimmune (predominantly autoimmune thyroiditis), and 10% had psychiatric diseases, respectively. Skin tumors were the most common malignant diseases (47% of all tumors), while hepatocellular carcinoma rarely occurred (only six cases). Overall long-term mortality and liver-associated mortality were 9.16% and 4.67%, respectively. However, long-term survival was strongly associated with disease remission. **Conclusions:** Although AIH is a silent disease and cirrhosis is present in many cases, a favorable long-term prognosis can be achieved by consequent immunosuppressive therapy. The incidence of (liver-associated) complications seems to be lower in comparison to other etiologies, such as viral hepatitis or NASH, and mainly depends on the long-term side effects of immunosuppressive therapy.

## 1. Introduction

Autoimmune hepatitis (AIH) is a complex and progressive inflammatory liver disease characterized by immune-mediated destruction of the liver parenchyma (interface hepatitis), hypergammaglobulinemia, and the presence of circulating autoantibodies [1,2]. Its prevalence is relatively rare and ranges from 16 to 18 cases per 100,000 inhabitants in Europe, but it is increasing in both women and men [3,4].

Due to the absence of specific diagnostic markers and the heterogenous spectrum of clinical, laboratory, and histological manifestations, AIH diagnosis can be difficult. Therefore, diagnostic criteria for AIH have been proposed in a simplified manner for routine clinical use with a scoring system (the simplified diagnostic criteria of the International Autoimmune Hepatitis Group) [5,6].

To date, the etiology of AIH is still unknown, and other causes of acute or chronic liver disease must be excluded in order to diagnose AIH. It is hypothesized that AIH develops in individuals with a certain genetic background due to environmental factors (triggering factors such as drugs or biological agents), suggesting that they have awakened latent autoimmunity [7,8,9]. This assumption is underlined by strong genetic associations within genes of the human leukocyte antigen (HLA) region (the human major histocompatibility complex, MHC), which are involved in the presentation of antigenic peptides to T cells and are therefore implicated in the initiation of an adaptive immune response [10]. There are particularly strong associations with the HLA DR3 and DR4 molecules conferring susceptibility to AIH type 1 in Europe and North America [11,12].

Furthermore, AIH is characterized by a favorable response to immunosuppressive therapy. In contrast, if the disease goes untreated, it will often lead to cirrhosis, liver failure, and death [5]. The mainstay of AIH treatment includes corticosteroids alone or in combination with azathioprine to achieve normalization of transaminases and immunoglobulin G (IgG) levels in serum. To reduce steroid-specific side effects, the topical steroid budesonide can be used in non-cirrhotic patients instead of prednisolone. If the standard therapy fails, immunomodulatory drugs such as mycophenolate mofetil, tacrolimus, cyclosporine A, or rituximab can be applied in order to achieve disease remission [12,13,14,15,16].

However, data on the clinical course and the long-term outcome of patients suffering from AIH are scarce. Here we report our experience with a large single-center cohort with patient data collected over a span of 20 years comprising 535 AIH patients. The data include demographic characteristics, laboratory profiles, immunosuppressive therapy, clinical course, and long-term outcome.

## 2. Patients and Methods

### 2.1. Patient Information, Data Collection, and Ethical Considerations

In this retrospective study between 2000 and 2020, a total of 535 patients with AIH were included. The patient data were collected on the basis of ICD-10 codes (K75) from the hospital information system (HIS) and digital archive (MEDICO KIS) of the University Hospital Essen. AIH was diagnosed according to the “Simplified Diagnostic Scoring System of the International Autoimmune Hepatitis Group” including the analysis of auto-antibodies, immunoglobulins, viral markers, and histological findings if available [6,17,18,19]. Liver histology was obtained in all seronegative patients either by percutaneous- or laparoscopy-guided biopsy. If patients had at least 6 points in the simplified AIH score, histology was not mandatory. The University Clinic of Essen ethics committee approved the retrospective, anonymous analysis of the data, and the study was conducted according to the principles expressed in the Declaration of Helsinki. All patients gave their written informed consent prior to study inclusion.

### 2.2. Laboratory Parameters, Immunosuppressive Therapy, and the Definition of Therapy Response

At initial presentation, liver enzymes, total bilirubin, serum creatinine, INR, albumin, alpha-fetoprotein (AFP), IgG, ɣ-globulins, antibody profiles (ANA, AMA, ANCA, SMA, LKM, and SLA), viral markers, and HLA loci, if available, were analyzed. Autoantibodies were tested by enzyme-linked immunosorbent assay (ELISA) and indirect immunofluorescence technique (IFT). After the diagnosis of AIH, the patients received steroid pulse therapy (prednisolone 1–2 mg/kg body weight or budesonide 9 mg) with consecutive down-tapering to 0–7.5 mg prednisolone (0–3 mg budesonide) daily as maintenance therapy. Further immunosuppressive therapy was conducted in dependence on the steroid response and patient’s clinical profile using immunomodulatory drugs, predominantly azathioprine (1–1.5 mg/kg body weight/day).

*Complete biochemical response* was defined as the normalization of alanine aminotransferase (ALT) (<50 IU/L in male patients, <35 IU/mL in female patients) and IgG levels within 6 months after initiation of treatment according to the International Autoimmune Hepatitis Group (IAIHG). An *insufficient response* by 6 months was a failure to meet the above definition. *Non-response*, defined as <50% decrease of serum transaminases within 4 weeks after the initiation of treatment, was not analyzed in our study. However, data on the *remission* defined as liver histology with a Hepatitis Activity Index <4/18 according to the IAIHG could not be determined since a follow-up liver biopsy was not routinely performed. *Intolerance to treatment* was defined as any adverse event possibly related to treatment leading to drug discontinuation [20].

### 2.3. Statistical Analysis

Statistical analysis was performed using GraphPad Prism, version 6.00 for MacOsX (GraphPad Software, San Diego, CA, USA). For descriptive statistics, the medians and IQR were determined. All variables were tested for normal distribution with the Kolmogorov–Smirnov test, the Shapiro–Wilk test, and the calculation of skew and kurtosis. The Mann–Whitney U test was used to compare differences between independent groups. Categorical data were tested with the chi-square test, and the Kruskal–Wallis test was used for multiple comparisons. A *p* value < 0.05 was considered statistically significant.

## 3. Results

### 3.1. Demographic and Laboratory Data

Between the years 2000 and 2020, a total of 535 adult patients with autoimmune hepatitis (AIH) and a well-documented long-term course were included in this retrospective study. The majority of the study population was female (*n* = 401, 74.95%), the mean age at initial diagnosis was 44.84 [range 18–81] years, and the mean observation period was 78.14 months. A total of 101 out of 535 patients (18.88%) presented with acute fulminant hepatitis or acute liver failure (ALF). ALF was defined as acute liver injury in individuals without a prior liver injury resulting in coagulopathy (international normalized ratio (INR) > 1.5) and any grade of hepatic encephalopathy (definition of the *Acute Liver Failure Study Group Germany*) [21]. Acute fulminant hepatitis was defined as high transaminases > 1′000 U/mL, hyperbilirubinaemia > 5 mg/dL, and massive necrosis of the liver parenchyma in histology without fulfilling the criteria for ALF. Histology was not available for 189/535 patients (35.33%). Demographic and laboratory data on the initial diagnosis are presented in Table 1.

### 3.2. Autoantibodies and Genetic Background

Positive values were found in ANA 72.52% (*n* = 388), SMA 16.84% (*n* = 90), AMA 8.04% (*n* = 43), SLA 6.17% (*n* = 33), LKM 2.43% (*n* = 13), and ANCA 2.24% (*n* = 12), respectively. However, 147/535 patients were seronegative (27.5%). Accordingly, the vast majority of patients were classified into type 1 AIH (*n* = 522/535; 97.57%).

Genetic determination of HLA epitopes was available for 204/535 patients (38.13%). Prevalence for the different HLA loci were as follows: HLA-A*01 40.19% (*n* = 82), HLA-B*08 38.23% (*n* = 78), HLA-DRB*03 42.16% (*n* = 86), and HLA-DRB*04 36.27% (*n* = 74), respectively. Most patients were positive for multiple HLA epitopes (predominantly the combination of A*01, B*08, and DRB*03), while 46 patients were negative for all tested HLA loci (22.55%). The HLA DRB107 allele predisposing to AIH2 was not routinely tested in our collective.

### 3.3. Overlap to Other Autoimmune Diseases and Co-Morbidities

**Figure 1:**
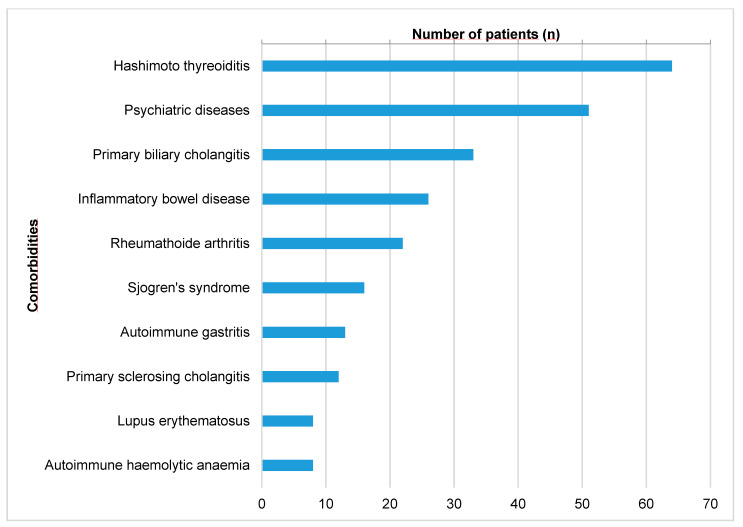
Concomitant (autoimmune) diseases in AIH patients (*n*).

The patients were screened for concomitant autoimmune diseases at first diagnosis and in case of clinical suspicion during the course. Autoimmune thyroiditis was the most common overlapping autoimmune disease affecting 64/535 (11.96%) AIH patients. Chronic cholestatic liver diseases were present in 55/535 (8.41%) AIH patients (primary biliary cholangitis (PBC) 6.17% and primary sclerosing cholangitis (PSC) 2.24%). Furthermore, 38/535 patients (7.10%) suffered from concomitant inflammatory rheumatic diseases (22 rheumatoid arthritis, 16 Sjögren’s syndrome (thereof 5 with sicca syndrome), and 26/535 patients (4.86%) from inflammatory bowel diseases (18 ulcerative colitis, 12 Crohn’s disease). Concomitant autoimmune diseases in patients with AIH are shown in Figure 1. Interestingly, 51/535 patients (9.53%) with AIH had concomitant psychiatric diseases with depressive symptoms.

### 3.4. Immunosuppression and Therapy Response

**Figure 2:**
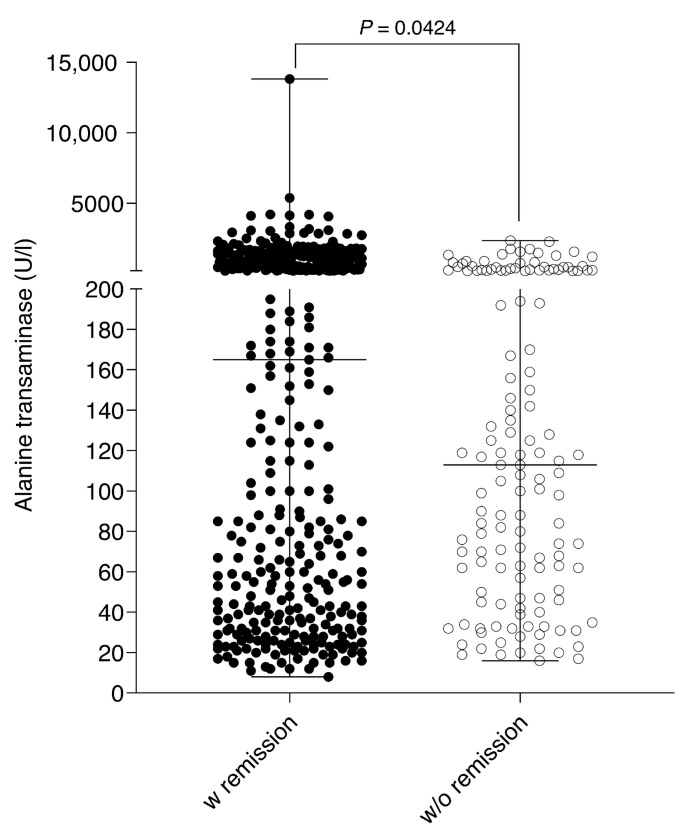
Relationship between serum ALT levels and remission.

A total of 417/535 (77.94%) patients were treated with high-dose steroids due to acute AIH (prednisolone 1–2 mg per kg per day or budesonide 9 mg per day) as induction therapy, which was then slowly tempered down by scheme to a low-dose maintenance therapy or completely withdrawn. If steroids were given over a time period of >8 weeks, vitamin D prophylaxis was administered. For maintenance therapy, azathioprine was the first-line treatment (*n* = 380/535, 71.03%). By use of this scheme, 315/535 (58.88%) patients attained *complete biochemical responses* according to the definition of the IAIHG. In case of azathioprine *intolerance* (20/380; 5.26%) or *insufficient response* (40/380; 10.53%), immunosuppressive therapy was switched to second-line immunomodulatory drugs such as 6-mercaptopurine (first second-line alternative), mycophenolic acid (second second-line alternative), ciclosporin A (third second-line alternative), or tacrolimus (fourth second-line alternative). The CD20 antibody rituximab was administered in only two patients (0.37%) refractory to first- and second-line therapies. By use of pharmacological therapies, a total of 397/535 (74.21%) patients achieved sustained biological and clinical remission. However, 138/535 patients (25.79%) were refractory to any therapy and did not achieve a remission state. Interestingly, higher ALT levels at initial presentation were associated with higher remission rates (ALT remission 163,5 U/l vs. ALT no remission 111 U/l, *p* = 0.0424, Figure 2).

### 3.5. Tumor Diseases

**Figure 3:**
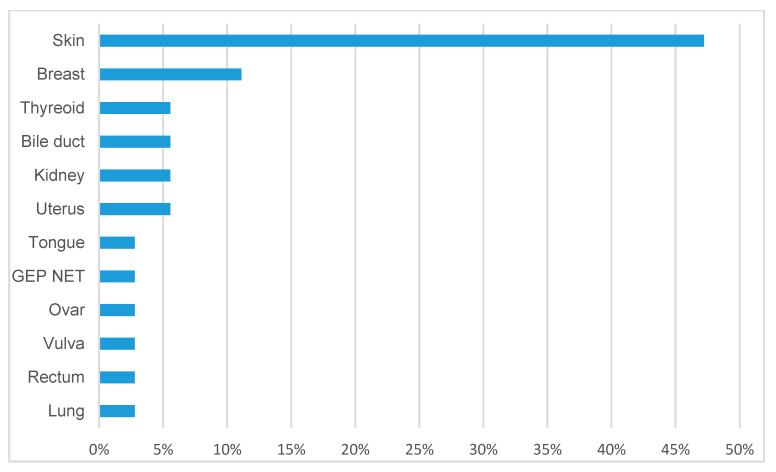
Malignant tumor diseases of 535 AIH patients (% of all malignant diseases).

A total of 42/535 patients (7.85%) developed malignant neoplasia during the course of the study. Skin tumors (melanoma, squamous cell carcinoma, and basal cell carcinoma; *n* = 17; 3.18%) were the most common malignant neoplasia, followed by hepatocellular carcinoma (HCC; *n* = 6; 1.12%), and breast cancer (*n* = 4). Malignant skin tumors were associated with azathioprine intake (*n* = 15; 88.26%). All patients with HCC suffered from liver cirrhosis. Malignant tumor diseases of AIH patients are demonstrated in Figure 3.

### 3.6. Long-Term Outcome and Mortality

**Figure 4:**
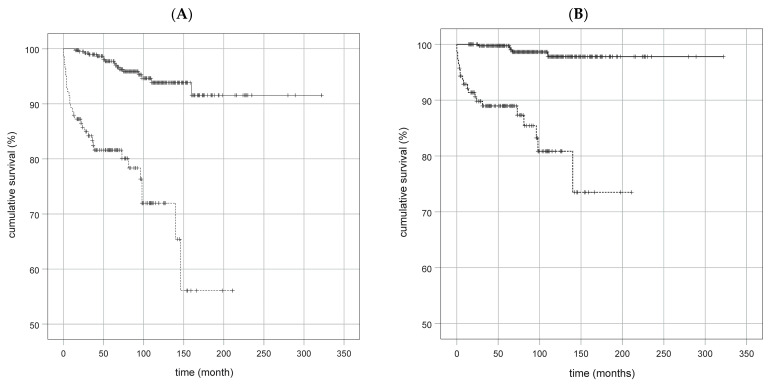
(**A,B**): Outcomes of AIH patients w/wo remission. (**A**): general mortality: 20-year survival of 91.6% with remission vs. 56% without remission. (**B**): liver-associated mortality: 20-year survival of 97.8% with remission vs. 73.5% without remission. *p* < 0.0001 (both); continuous line = with remission, intermittent line = without remission.

A total of 169/535 patients (31.59%) presented with liver cirrhosis due to AIH at initial diagnosis. Diagnosis of liver cirrhosis was based on clinical, laboratory, imaging, transient elastography (Fibroscan), and/or histological findings. Overall mortality was 9.16% (49/535 patients), while liver-associated mortality was 4.67% (25/535 patients). Mortality was strongly associated with disease remission (complete biochemical response), which is demonstrated in Figure 4A,B. In addition, mortality was increased when concomitant chronic inflammatory bowel disease was present (10-year survival AIH with or without Crohn’s disease 83% vs. 94%, *p* = 0.028; 10-year survival AIH with or without ulcerative colitis 76% vs. 94%, *p* = 0.026).

## 4. Discussion

We herein report our long-term experience of more than 500 AIH patients for an observational period of 20 years. To the best of our knowledge, this is the largest single-center study pertaining to AIH so far. Although AIH can affect all age groups and both sexes, the “typical” AIH patient in our collective was a middle-aged female (75% women, mean age 45 years) with AIH type 1 (97%), which matches with epidemiological data according to the current literature (mean age: 29–56 years) [22,23,24,25,26,27,28,29,30,31,32,33,34].

Conventional AIH-specific autoantibodies are key to the diagnosis of AIH. However, these antibodies in serum were absent in almost 30% of cases within our collective, a fact which underlines the need for other diagnostic tools in our armamentarium. Histological examination by liver biopsy still represents the “gold standard” of the diagnostic work-up in patients with suspected AIH. However, various histological findings associated with AIH are non-specific and present in other cases of, for example, viral hepatitis or drug-induced liver injury, which confounds the interpretation of these findings [5,35]. It is known that individuals with certain human leukocyte antigen (HLA) haplotypes are more susceptible to the development of AIH. These alleles are located on the short arm of chromosome 6 within the region of DRB-1 [10,11,12]. In our cohort, AIH patients were positive for HLA HLA-A*01 in 40.19%, HLA-B*08 in 38.23%, HLA-DRB*03 in 42.16%, and HLA-DRB*04 in 36.27%, respectively. When comparing our data to the HLA haplotypes registry data from 20 million healthy individuals, statistical significance was reached for HLA-A*01 (*p* = 0.01), HLA-B*08 (*p* = 0.001), and finally, HLA-DRB*03 (*p* < 0.001) [36]. Thus, the determination of these HLA haplotypes might be useful to establish a diagnosis of AIH, particularly in uncertain cases.

AIH is associated with a wide variety of other autoimmune or immune-mediated diseases. In accordance, concomitant autoimmune diseases in patients with AIH are shown in Figure 1. Approximately 12% of patients suffered from autoimmune thyroiditis and 7% from inflammatory rheumatic diseases. Thus, in accordance with actual guidelines, an extended diagnostic screening for other autoimmune diseases is recommended at diagnosis [5,14,32,37,38].

Interestingly, approximately 10% of AIH patients in our collective had concomitant psychiatric diseases with depressive symptoms. This matches with data from the current literature, where depressive syndrome occurred in 10.8% and was found to be five times more frequent in AIH patients compared to the general population [39]. In addition, several studies have shown that severe symptoms of depression and anxiety negatively impact the quality of life in AIH patients [39,40,41]. Therefore, detailed psychiatric assessment and complementary psychological treatment offer the chance to improve care and should therefore be offered to AIH patients.

AIH can be asymptomatic or present in various forms, from subclinical disease to acute liver failure and end-stage liver disease, as represented by our collective. Since AIH often proceeds as a chronic inflammatory and silent disease, the presence of advanced fibrosis or liver cirrhosis at first diagnosis is not uncommon. The presence of cirrhosis at diagnosis is, however, correlated with a negative outcome (liver transplantation or death). In our cohort, approximately 32% of patients were diagnosed with cirrhosis due to AIH, which matches with data from the current literature. In a cohort of 450 AIH patients, Werner et al. reported that 30% had evidence of cirrhosis at first diagnosis, with a further 10% developing cirrhosis during a median follow-up time of 7.2 years [38]. Accordingly, Feld and colleagues demonstrated that 33% of patients had histological proof of cirrhosis [42]. In coherence with the current literature, cirrhosis rates due to AIH at diagnosis are high and vary between 13 and 41% [24,25,26,27,28,29,30,31,32,33,34].

A key factor in improved prognosis in AIH is obtaining disease remission. The dynamic reaction toward chronic hepatocyte injury (inflammation) resulting in increasing fibrosis has to be interrupted to prevent disease progression. There is solid evidence supporting the fact that liver fibrosis (or even cirrhosis) is potentially reversible as long as a certain “point of no return” is not exceeded [43]. Corticosteroids (prednisolone or topical budesonide for induction) in combination with azathioprine (for maintenance) is the first-line treatment; clinical and biochemical remission is thereby achievable in up to 90% of cases. In case of treatment failure, incomplete response, drug intolerance, or relapse, alternative treatment strategies are needed. However, no randomized controlled trials have been performed for second-line options. Mycophenolate mofetil is the most widely used second-line drug and has good efficacy, particularly for patients intolerant to azathioprine, but it has the major disadvantage of being teratogenic. Only scarce and inconsistent data on calcineurin inhibitors and tacrolimus are available. In addition, experience with the CD-20 antibody rituximab has been published for exceptional and refractory cases [35,44,45]. Among our cohort, 74% of patients achieved sustained biological and clinical remission, predominantly by the use of steroids in combination with azathioprine. However, a high response rate to immunosuppressive therapy is considered to be characteristic of AIH and was reached in 60–100% of cases [24,26,27,28,29,30,31,33,34]. At least 138/535 patients (25.79%) were refractory to any therapy and did not achieve a remission state, which was associated with worse outcomes/increased mortality. This substantial number of patients possibly does not mirror a “daily clinical routine” since the University Hospital Essen is a referral center for complex liver diseases. In particular, a significant number of patients were not therapy naïve at initial presentation. Interestingly, disease remission was positively correlated with inflammatory activity, meaning the higher the GPT levels at diagnosis, the higher the remission rate (Figure 2).

The development of malignant tumor diseases is demonstrated in Figure 3. The vast majority were tumors of the skin (nearly 50% of all tumors), e.g., melanoma, squamous cell carcinoma, and basal cell carcinoma, respectively. Skin cancer is a well-defined complication of immunomodulatory drugs, particularly azathioprine [46,47,48]. Patients treated with these agents should, therefore, routinely be monitored by a dermatologist. Hepatocellular carcinoma (HCC), as a complication of chronic liver disease, is one of the most common malignancies worldwide, and its incidence is rising. Most HCCs develop on the basis of liver cirrhosis (>90%) but can in rare cases also arise in non-cirrhotic livers. Although some studies report on HCC occurrence for patients with AIH, the risk for HCC seems to be substantially lower compared to other entities such as, for example, viral hepatitis or non-alcoholic steatohepatitis. In their review, including 11 studies with 8460 patients between 1989 and 2016, Valean and colleagues reported HCC occurrence rates for AIH patients with cirrhosis from 0.2 to 12.3%, while the proportion of non-cirrhotic HCCs was estimated at 1.03% [49]. In addition, Teufel et al. analyzed 431 patients with AIH and found 3 HCCs, all of them had liver cirrhosis [50]. Our analysis confirms these previously published data. HCC incidence was rare with 1.12% throughout the whole study population, while all HCCs occurred in cirrhotic patients (HCC incidence in AIH-induced cirrhosis was 3.6%). None of the non-cirrhotic AIH patients were diagnosed with or developed HCC.

The overall goal of AIH treatment is to induce and maintain complete suppression of inflammatory activity and to prevent disease progression to liver cirrhosis and decompensation. The mainstay of AIH treatment contains an induction phase and a maintenance phase. Per definition, remission is achieved when (i) clinical symptoms are absent and (ii) transaminases (and immunoglobulins) have returned to normal ranges [51,52]. Standard induction therapy is performed with high-dose prednisolone (or budesonide in the absence of cirrhosis) with a dose of 1–2 mg/kg/day, while azathioprine is usually used for maintenance therapy. Since the azathioprine effect lasts up to 4–6 weeks, therapy initiation is usually performed simultaneously with steroid treatment [5,15,16,45]. In case of treatment failure or relapse, alternative immunosuppressive agents such as mycophenolate mofetil, tacrolimus, or cyclosporine A can be employed. However, prospective trials are missing, and evidence is mainly based on expert opinion [5,6,12]. Liver transplantation is the ultimate rescue therapy for all liver diseases, but fortunately has only a minor role in AIH [12,53]. In comparison to other etiologies, the long-term prognosis of AIH is favorable and appears to be most dependent on the presence of cirrhosis at diagnosis and initial treatment response [27,54]. According to Gleeson et al., who summarized 11 studies of long-term mortality in AIH, mortality rates over 10 and 20 years were median 13% (range 5–26%) and 31% (range 18–53%), respectively [54]. Among our patient collective, overall long-term mortality and liver-associated mortality were 9.16% and 4.67%, respectively. However, long-term survival was strongly associated with disease remission (Figure 4A,B). In addition, mortality was increased when concomitant chronic inflammatory bowel disease was present (10-year survival AIH with (83%) or without (94%) Crohn’s disease, *p* = 0.028; 10-year survival AIH with (76%) or without (94%) ulcerative colitis, *p* = 0.026).

We are aware of the limitations of our study, the most important of them being a retrospectively performed single-center study.

In summary, we herein reported on our long-term experience with a large single-center cohort of AIH patients. Although AIH is a silent disease and advanced CLD or cirrhosis is present in many cases, a favorable long-term prognosis can be achieved by consequent immunosuppressive therapy. The incidence of (liver-associated) complications appears to be lower in comparison to other etiologies, such as viral hepatitis or NASH, and mainly depends on the long-term side effects of immunosuppressive therapy.

## Figures and Tables

**Table 1:** Demographic and laboratory data on initial diagnosis.

Parameter	Value	Reference Value
Mean age (years)	44.8 (18–81)	
Male	134 (25%)	
Female	401 (75%)	
BMI (kg/m^2^)	25.63 (16.1–46.6)	18.5–24.9
INR	1.07 (0.99;1.28)	
Total Bilirubin (mg/dL)	1.2 (0.7;6.3)	0.3–1.2
ALT (U/L)	132.5 (46;712)	<35
Cholinesterase (U/L)	5.7 (3.9;7.8)	4.9–11.9
Albumin (%)	54.8 (49;59.9)	55.8–66.1
Gamma-Globulin (%)	21 (16.2;27.6)	11.1–18.8
Immunoglobulin G (mg/dL)	15.8 (12;21.1)	7–16
AIH type 1	522 (97.6%)	
AIH type 2	13 (2.4%)	

## Data Availability

Data will be made available by the corresponding author upon reasonable request.

## Abbreviations

AFP, alpha-fetoprotein; AIH, autoimmune hepatitis; ALF, acute liver failure; ALT, alanine aminotransferase; ANA, antinuclear antibodies; ANCA, anti-neutrophil cytoplasmic antibody; CsA, cyclosporin A; CNI, calcineurin inhibitor; DILI, drug-induced liver injury; HCC; hepatocellular carcinoma; HLA, human leukocyte antigen; IAIHG, International Autoimmune Hepatitis Group; IgG, immunoglobulin G; INR, international normalized ratio; LKM, anti-liver kidney microsomal antibody; LT, liver transplantation; MELD, model of end-stage liver disease; MMF, mycophenolate mofetil; PBC, primary biliary cholangitis; PSC, primary sclerosing cholangitis; SLA, anti-soluble liver antigen; SMA, anti-smooth muscle antibody.
